# Leontiasis ossea and post traumatic cervical cord contusion in polyostotic fibrous dysplasia

**DOI:** 10.1186/1746-160X-2-24

**Published:** 2006-08-15

**Authors:** Boby Varkey Maramattom

**Affiliations:** 1 Department of Neurology, Lourdes Hospital, Kochi, Kerala, India

## Abstract

Leontiasis ossea (leonine facies) or cervical canal stenosis are rare complications of polyostotic fibrous dysplasia (PFD). This case report documents dramatic leontiasis ossea in PFD as well as post traumatic cervical cord contusion due to hyperextension injury in a patient with generalized PFD involving the cranio-facial bones, axial skeleton and entire spine with secondary cervical canal stenosis. Cervical cord contusion has not been reported earlier in PFD.

## Background

Fibrous dysplasia (FD) is a rare skeletal developmental disorder whereby the medulla of bone is replaced by fibrous tissue leading to distortion of bony architecture, expansion and weakening of bones, easy fractures, joint subluxations or dislocations and compressive symptoms [1]. Four varieties of FD are recognized; the monostotic form (single bone involvement), polyostotic form (PFD) [multiple bones are affected], craniofacial form (multiple craniofacial bones are affected) or a cherubic form (maxilla and mandible alone are affected). PFD has a predilection for the long bones, ribs, spine and craniofacial bones. It is sometimes associated with the McCune Albright syndrome where café au lait spots and endocrinopathies (particularly precocious puberty) coexist. Rarely, other endocrine dysfunction such as hyperthyroidism, growth hormone excess, Cushing syndrome or primary hyperparathyroidism can also be associated with PFD. Although FD is linked to an activating mutation in the gene that encodes the subunit of stimulatory G protein (Gs) located at 20q13.2–13.3, it is a non-heritable congenital developmental disorder.

Fibrous dysplasia predisposes the spine to atlanto-axial instability [2], odontoid fractures [3], compression fractures, spinal cord compression via expansile lesions [4,5] sarcomatous transformation [6] or scoliosis [7]. Although PFD can produce spinal canal stenosis with consequent pathological implications, cervical cord contusions have never been reported before with this disorder.

I would like to report a case of PFD affecting the entire spine, producing cervical canal stenosis and post traumatic cervical cord contusion. This report is also noteworthy for its dramatic depiction of leontiasis ossea, a peculiar facial deformity sometimes associated with PFD.

## Case report

**Figure 1:**
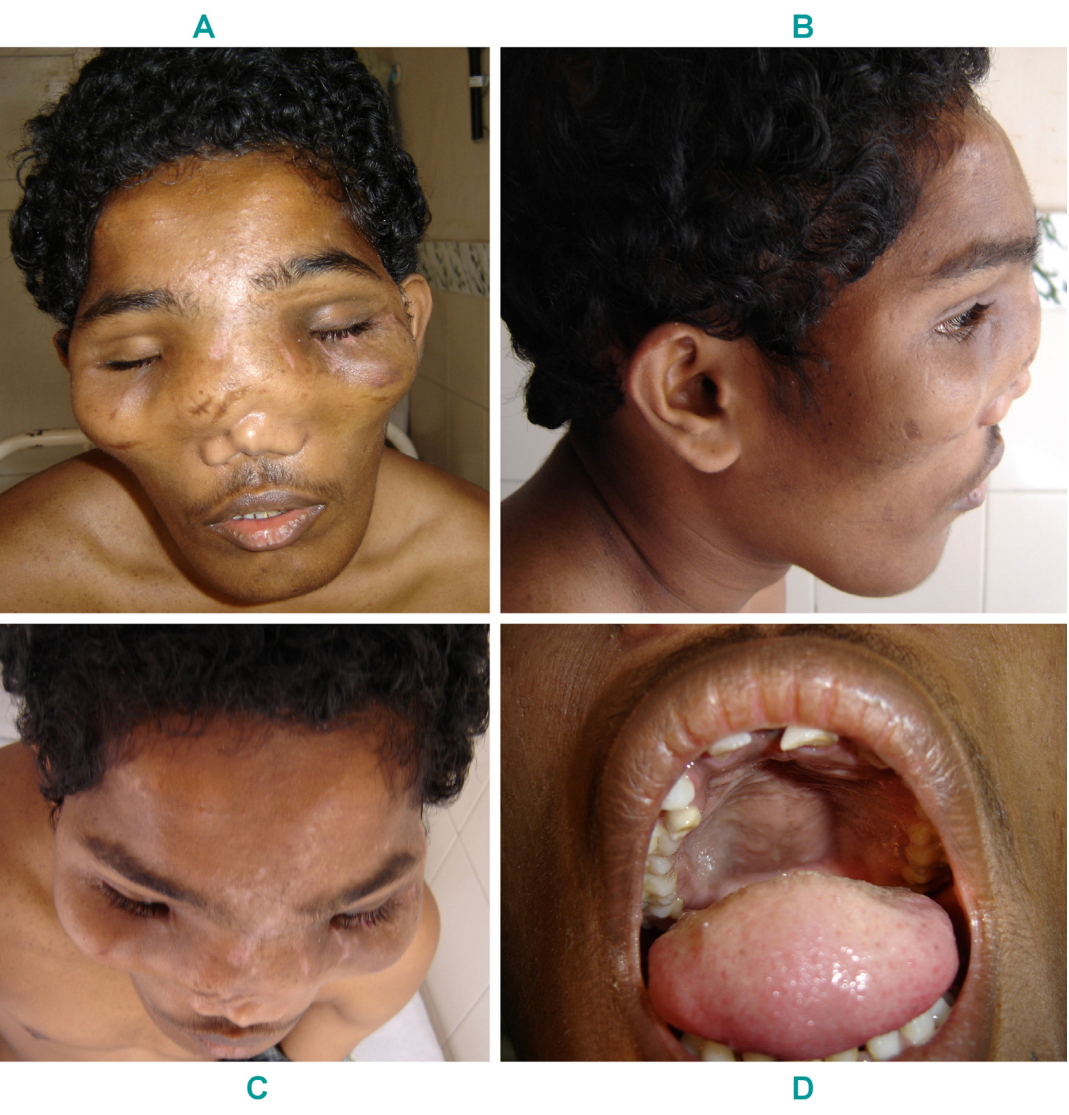
Leontiasis ossea. Panels A–C showing frontal and maxillary bossing. Panel D showing hard palate swelling.

**Figure 2:**
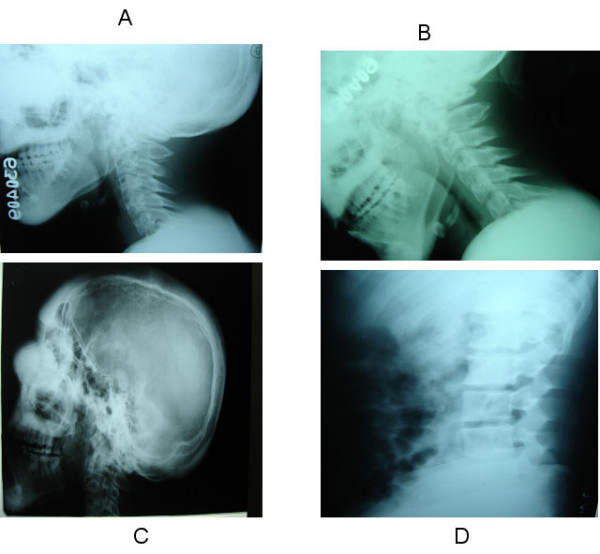
Panel A and B show the cervical spine Xray in neutral position and flexion respectively. Note the hypertrophy of the posterior elements. Panel C shows diffuse cortical thickening on a lateral skull X-ray with soft tissue thickening and elevation of the nasal bridge. Panel D shows diffuse thickening of the posterior elements of the lumbar vertebrae.

A 25 year old man was brought to the emergency room (ER) with quadriparesis after a fall from a bicycle. Although he had facial deformities since early childhood, he professed only to cosmetic embarassment, nasal blockage, mouth breathing, mild snoring and progressing bowing of his shins. There were no other affected family members. He was gainfully employed and had been riding a bicycle to work, when he skidded off the road into a sand patch, landing on his face. At the point of impact, he bruised his face. He was able to turn over, but immediately noticed some neck pain and weakness of all four limbs. He was just able to lift his arms and move his legs from side to side. He was transferred by ship from his island to our hospital and was seen three days after the injury. On examination, he had leontiasis ossea (leonine facies due to symmetrical frontal and maxillary bossing) (Figure 1). Examination of the oral cavity showed a symmetric soft tissue bulge of the hard palate (Figure 1). He had broad forearms, sausage like fingers and bowing of both legs with saber shins (Figure 2). The nasal bridge was flat and elevated due to a soft tissue thickening (Figure 1 &2). No café au lait spots were observed. Neurological examination revealed grade 3 power (MRC scale) in his arms with pronounced distal hand muscle weakness and grade 2 power in his legs. All deep tendon reflexes were absent, plantar reflexes were extensor and there was a sensory level to all modalities at the groin.

**Figure 3:**
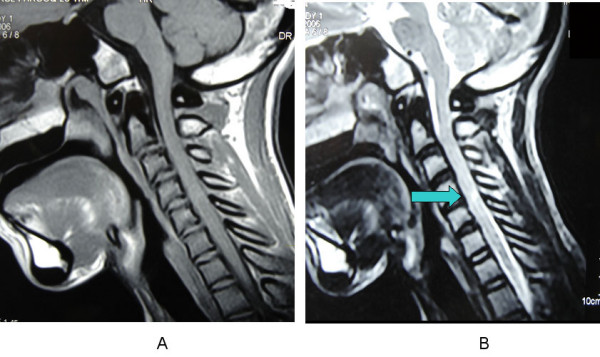
MRI composite Sagittal T1 weighted MRI image showing enlargement and hyperintensities involving the posterior elements of the cervical vertebrae. Sagittal T2 weighted MRI image showing a cervical cord contusion at C 4–5 level (Green arrow).

MRI of the spine showed diffuse enlargement of the laminae, transverse processes and spinous processes of the entire spine (mainly involving the posterior elements) with cervical canal stenosis and a cervical cord contusion at C 4–5 level. On T1 weighted images the posterior elements were hyperintense. The facet joints of the cervical vertebrae were also enlarged. (Figure 2) The cranio-vertebral junction was normal. Skull X-rays and CT scans (Figure 3) with bone windows showed diffuse thickening of the inner and outer tables of all the skull bones with a widened diploe. The brain parenchyma, subarachnoid cisterns and paranasal sinuses were normal. His routine blood examinations, blood glucose, serum calcium, phosphate, alkaline phosphate levels, and thyroid function tests were normal. Urinary mucopolysaccharides were absent. The clinico-radiological picture was compatible with a generalized form of PFD. He was initiated on IV methylprednisolone 1 gm OD for 5 days and was able to start walking in 5 days. He was discharged home on the 10^th ^day with residual distal hand muscle weakness and grade 4 muscle power in his legs.

## Discussion

Cervical canal stenosis (CCS) predisposes patients to cervical cord injury after traumatic hyperflexion or hyperextension movements. The cervical cord is most mobile between the C3–C6 segments and nearly fills the spinal canal at this level. Hence these segments bear the brunt of injury during cervical spine trauma and patients can develop neuropraxia, cord contusions, hematomas or cord transections. CCS may be developmental or acquired due to a number of causes such as cervical spondylosis, diffuse idiopathic skeletal hyperostosis, ossification of the posterior longitudinal ligament (OPLL) or rheumatoid arthritis.

PFD is a rare cause of CCS because it afflicts the lumbar, thoracic, sacral and cervical vertebrae in descending frequency. Nearly 70% of the lesions involve only the posterior aspects of the spine [8]. The most common abnormality seen in PFD is scoliosis (~50% of patients). PFD has rarely been reported to cause pathologic compression fractures of the lumbar spine [9]. Our case is remarkable in that PFD involved the entire vertebral column producing cervical canal stenosis predisposing the patient to cervical cord contusion during hyperextension injury. Another remarkable feature was the leontiasis ossea involving the entire craniofacial skeleton which has rarely been reported [10].

Although the term 'leontiasis ossea' is widely used for localized swellings of the face including those involving the jaw, it should be restricted to a generalized homogenous swelling that implicates most facial bones [11]. True leontiasis ossea is a rare facial deformity that is encountered in polyostotic FD, Albright's syndrome and rarely with Paget's disease, uremia with secondary hyperparathyroidism or acromegaly. Leontiasis ossea can be associated with progressive proptosis, visual impairment or nasal obstruction. Our patient had a relatively asymptomatic leontiasis ossea of long duration with only mild obstructive nasal symptoms, snoring and cosmetic disfigurement.

In conclusion, we present an unusual presentation of PFD with diffuse involvement of the cranio-facio-vertebral skeleton with leontiasis ossea and a post traumatic cervical cord contusion secondary to hyperextension injury of the cord in a compromised cervical canal.

## Competing interests

The author(s) declare that they have no competing interests.

## Authors' contributions

The author was wholly responsible for all aspects of this study including data collection, writing up the paper and takes full responsibility for the integrity of the data and the accuracy of the analyses.

**Figure 4:**
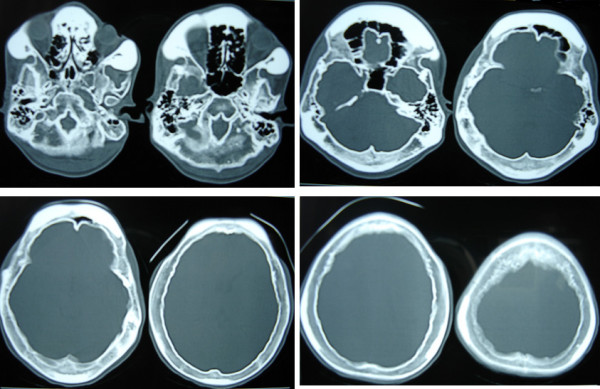
CT scan bone windows showing diffuse thickening of the skull bones with widening of the diploic space.
